# Investigation of Knowledge, Awareness, and Practices of Glaucoma in Riyadh City, Saudi Arabia

**DOI:** 10.7759/cureus.51700

**Published:** 2024-01-05

**Authors:** Dania Bamefleh, Othman M Alassaf, Zaid M Algahtani

**Affiliations:** 1 Department of Glaucoma, King Khaled Eye Specialist Hospital, Riyadh, SAU; 2 Department of Ophthalmology, King Khalid Hospital, Hail, SAU; 3 College of Medicine, King Saud Bin Abdulaziz University for Health Sciences, Riyadh, SAU

**Keywords:** saudi arabia, riyadh region, public awareness, disease awareness, glaucoma

## Abstract

Background

Glaucoma is a group of conditions characterized by progressive irreversible optic neuropathy, intraocular pressure as a modifiable risk factor, and, in many patients, visual field defects. Glaucoma has recently been classified as a neurodegenerative disorder of the optic nerve that results in the loss of retinal ganglion cells. This study evaluated levels of awareness of glaucoma in the general population of Riyadh City, Saudi Arabia.

Methodology

A pre-designed, pre-validated online questionnaire from previous studies was distributed to respondents via online social media platforms. Factors associated with awareness of glaucoma were analyzed statistically.

Results

Responses were received from 585 participants aged ≥18 years, including 309 (52.8%) men and 276 (47.2%) women. Gender, a diagnosis of glaucoma, a positive family history of glaucoma, and undergoing glaucoma screening were significantly associated with the level of glaucoma knowledge (p < 0.05 each). Age group (p = 0.587) and level of education (p = 0.136) were not significantly associated with the level of glaucoma knowledge.

Conclusions

The population of Riyadh City exhibited a low level of glaucoma awareness, indicating an urgent need for comprehensive ophthalmic health education initiatives aimed at enhancing public consciousness and awareness of glaucoma risks and complications.

## Introduction

Glaucoma is a set of diseases characterized by progressive, irreversible optic neuropathy, elevated intraocular pressure as a modifiable risk factor, and visual field defects [1]. Glaucoma has recently been classified as a neurodegenerative disorder of the optic nerve that results in the progressive loss of retinal ganglion cells [2]. In 2010, the World Health Organization reported that glaucoma was the second most common cause of blindness globally, after cataracts [3]. Because glaucoma has no distinct symptoms or signs during onset or the period before its clinical manifestation, patients frequently present with later-stage disease [4]. Glaucoma-related visual disability can have adverse effects on an individual’s social and economic well-being [5].

It has been reported that about one-third of glaucoma patients progress to blindness before seeing doctors or receiving medical care [6]. Late presentation is generally due to a lack of awareness and knowledge about the disease and its symptoms, with patients often ignoring early signs of this condition and failing to seek treatment at the appropriate time [7,8]. Early diagnosis and management are crucial in preventing the progression of glaucoma to blindness. National screening programs are needed, and the general population should be made aware of the importance of glaucoma and encouraged to seek regular ophthalmic care. Increased awareness of this condition and its consequences can encourage large numbers of individuals to participate in glaucoma screening programs, thus reducing the burden of glaucoma in populations [9]. The present study was designed to evaluate levels of awareness of glaucoma in the general adult population of Riyadh City, Saudi Arabia.

## Materials and methods

Ethics approval

The protocol of this study was approved by the Institutional Review Board of King Khaled Eye Specialist Hospital, Riyadh, Saudi Arabia (reference number: RD/26001/IRB/0446-23). Informed consent was obtained from all participants for participation and publication of the study. Participants were informed about the nature of the study and the confidentiality of their responses.

Data collection

This cross-sectional study was conducted in 2023 in the Riyadh region of Saudi Arabia, which is located in the center of the country and the center of the Arabian Peninsula and has a population of 7.7 million individuals. The General Authority for Statistics of the Kingdom of Saudi Arabia [10] has estimated that 4.08 million adults aged ≥18 years live in Riyadh City. The sample size was fixed at 585 participants and was calculated using the Raosoft equation. The parameters of this study included a 95% confidence interval, a 5% margin of error, and a p of 50% (to provide the maximum sample size). Participants were included if they were aged ≥18 years and were residents of Riyadh City, Saudi Arabia.

Data were obtained using an online questionnaire adapted from previous studies [11-13]. The questionnaire, which was composed of demographic questions and items related to awareness of glaucoma, was translated from English into Arabic and back-translated from Arabic into English to ensure its consistency. The questionnaire was distributed to participants via social media platforms, and a convenience sampling technique was used to include all eligible and consenting participants. The validity of the questionnaire was tested via a pilot study, with data from the pilot study excluded from the main study.

Data entry and analysis

The data collected were reviewed, coded, and inputted into SPSS Statistics for Windows version 22.0 (Released 2013; IBM Corp., Armonk, NY, USA). Statistical analyses were performed using Pearson’s chi-squared test, with statistical significance set at p < 0.05. For awareness and knowledge questions, each correct response was assigned a score of 1 point, with the maximum total score being 24 points. Scores of <6, 6-11, 12-17, and ≥18 points were regarded as indicative of very poor, poor, good, and excellent awareness, respectively. All variables, including demographic data, family history of glaucoma, and medical history of ophthalmic conditions, were subjected to descriptive analyses, with the results reported as frequencies and percentages. Levels of glaucoma awareness were assessed using frequency tables and figures. The relationships between participant awareness levels and their personal data and glaucoma screening were determined by cross-tabulation.

## Results

Participant characteristics

The present study included 585 respondents, 309 (52.8%) men and 276 (47.2%) women, with 425 (72.6%) aged 18-25 years. Most participants had a bachelor’s degree, 444 (75.9%), whereas 111 (19%) were high school graduates (Table 1). Of these individuals, 275 (47%) reported that their main sources of information about glaucoma were the Internet and social media, with 184 (31.5%) acquiring information from friends and relatives and 78 (13.3%) from physicians (Table 2).

**Table 1:** Demographic characteristics of the study population (N = 585). The data are represented as frequency (N) and percentage (%). Percentages are calculated based on the total number of participants (N = 585).

		Frequency (N)	Percentage (%)
Age group (years)	18–25	425	72.6%
	26–35	90	15.4%
	36–45	31	5.3%
	>45	39	6.7%
Gender	Male	309	52.8%
	Female	276	47.2%
Level of education	High school	111	19.0%
	Bachelor’s degree	444	75.9%
	Masters and‎/or PhD	30	5.1%

**Table 2:** Primary sources of information about glaucoma (N = 585). The data are represented as frequency (N) and percentage (%). Percentages are calculated based on the total number of participants (N = 585).

	Frequency (N)	Percentage (%)
Physician	78	13.3%
Social media	275	47.0%
Friends and‎/or relatives	184	31.5%
Books and/or literature	48	8.2%

Personal ophthalmic history and family history of glaucoma

Most participants, 407 (69.6%), did not have an ocular examination during the previous year. Ten (1.7%) participants reported being diagnosed with glaucoma, and 94 (16.1%) reported a family history of glaucoma. The most common ophthalmic condition was refractive error (n = 74), followed by dry eyes (n = 10) (Table 3).

**Table 3:** Personal ophthalmic history and family history of glaucoma (N = 585). The data are represented as frequency (N) and percentage (%). Percentages are calculated based on the total number of participants (N = 585).

		Frequency (N)	Percentage (%)
Have you undergone an ocular examination/screening in the past year?	Yes	178	30.4%
	No	407	69.6%
Have you been diagnosed with glaucoma?	Yes	10	1.7%
	No	575	98.3%
Do you have a family history of glaucoma?	Yes	94	16.1%
	No	491	83.9%
Do you have a history of an eye condition?	Refractive error	74	74.0%
	Dry eyes	10	10.0%
	Allergy	3	3.0%
	Uveitis	1	1.0%
	Cataract	3	3.0%
	Amblyopia	2	2.0%
	Refractive surgery	3	3.0%
	Strabismus	2	2.0%
	Keratoconus	2	2.0%

Glaucoma knowledge, awareness, and practices

Participants’ answers to the knowledge section of the questionnaire are summarized in Table 4. Only 221 (37.8%) reported that they had heard of glaucoma. When asked to describe the disease, 186 (28.7%) correctly indicated that glaucoma is a group of eye conditions that damage the optic nerve. When asked about the nature of glaucoma, 194 (33.2%) correctly indicated that it was associated with increased intraocular pressure, and 193 (33.0%) reported that vision could be affected during the early course of the disease. In addition, 249 (42.6%) correctly indicated that glaucoma could affect both eyes, 115 (19.7%) correctly indicated that glaucoma exhibited a familial predisposition, and 83 (14.2%) correctly indicated that early-stage glaucoma was generally asymptomatic. Although 260 (44.4%) of the participants correctly indicated that glaucoma is not the same as cataracts, only 145 (25.5%) responded correctly that glaucoma results from pressure damage to the optic nerve. In addition, 245 (41.9%) of the participants correctly indicated that, if left untreated, glaucoma could result in slow, irreversible loss of vision.

**Table 4:** Participant knowledge of glaucoma (N = 585). The data are represented as frequency (N) and percentage (%). Percentages are calculated based on the total number of participants (N = 585).

		Frequency (N)	Percentage (%)
Have you ever heard of glaucoma?	Yes	221	37.8%
	No	244	41.7%
	Maybe	120	20.5%
Glaucoma can be described as	A group of eye conditions that damage the optic nerve	168	28.7%
	A cloudy area in the lens of the eye	158	27.0%
	I do not know	259	44.3%
Glaucoma is related to increased intraocular pressure	Yes	194	33.2%
	No	23	3.9%
	Maybe	368	62.9%
Vision can be affected during the early course of glaucoma	Yes	193	33.0%
	No	71	12.1%
	Maybe	321	54.9%
Glaucoma is a condition that can affect both eyes	Yes	249	42.6%
	No	27	4.6%
	Maybe	309	52.8%
Glaucoma can exhibit familial predisposition	Yes	115	19.7%
	No	82	14.0%
	Maybe	388	66.3%
Early-stage glaucoma can have an asymptomatic course	Yes	83	14.2%
	No	157	26.8%
	Maybe	345	59.0%
Glaucoma is the same as cataracts	Yes	58	9.9%
	No	260	44.4%
	Maybe	267	45.6%
Glaucoma results from	Pressure damage to the optic nerve	149	25.5%
	Progressive increase in glasses power	4	0.7%
	Mature cataract	65	11.1%
	I do not know	367	62.7%
What happens if glaucoma is left untreated?	Slow, irreversible loss of vision	245	41.9%
	Eyes cannot be operated on	26	4.4%
	I do not know	314	53.7%

Although 261 (44.6%) participants correctly reported that the risk of glaucoma increases with age (Table 5), 283 (48.4%) indicated that people aged >50 years were at greater risk for this disease than those aged <50 years. In addition, 266 (45.5%) participants answered correctly that anyone can develop the disease, and 256 (43.8%) correctly indicated that glaucoma-related visual loss could be prevented, whereas only 94 (16.1%) correctly indicated that glaucoma-related visual loss cannot be reversed. Moreover, 106 (18.1%) responded that glaucoma was a hereditary disease, 153 (26.2%) stated that eye pain was a symptom of glaucoma, and 373 (63.8%) answered correctly that early screening could help avoid the complications of glaucoma. Of these, 296 (50.6%) correctly indicated that treatment of glaucoma was possible, and 396 (67.7%) reported that treatment modalities for glaucoma included medicated eye drops and ophthalmic surgery.

**Table 5:** Participant awareness of the characteristics of glaucoma (N = 585). The data are represented as frequency (N) and percentage (%). Percentages are calculated based on the total number of participants (N = 585).

		Frequency (N)	Percentage (%)
Risk of glaucoma increases with age	Yes	261	44.6%
	No	33	5.6%
	Maybe	291	49.7%
Anyone can develop glaucoma	Yes	266	45.5%
	No	43	7.4%
	Maybe	276	47.2%
Blindness from glaucoma can be prevented	Yes	256	43.8%
	No	28	4.8%
	Maybe	301	51.5%
Blindness resulting from glaucoma can be reversed	Yes	137	23.4%
	No	94	16.1%
	Maybe	354	60.5%
Glaucoma is a hereditary disease	Yes	106	18.1%
	No	76	13.0%
	Maybe	403	68.9%
People aged >50 years are more susceptible to glaucoma	Yes	283	48.4%
	No	25	4.3%
	Maybe	277	47.4%
Symptoms of glaucoma include eye pain	Yes	153	26.2%
	No	76	13.0%
	Maybe	356	60.9%
Early screening can help avoid the complications of glaucoma	Yes	373	63.8%
	No	16	2.7%
	Maybe	196	33.5%
Treatment of glaucoma is possible	Yes	296	50.6%
	No	28	4.8%
	Maybe	261	44.6%
Treatment modalities for glaucoma include	Medicated eye drops	59	10.1%
	Ophthalmic surgery	87	14.9%
	Both medicated eye drops and ophthalmic surgery	396	67.7%
	There is no treatment for glaucoma	43	7.4%

Participant awareness of glaucoma practices is shown in Table 6. Overall, 48% of the participants (n = 281) correctly indicated that the recommended time to start screening for glaucoma is after age 40 years. Although 178 (30.4%) of these participants reported having had an ocular examination during the previous year, only 25 (4.3%) had ever been screened for glaucoma. The mean ± standard deviation duration of eye symptoms before consulting a physician was 4 ± 2 days.

**Table 6:** Participant awareness of glaucoma practices (N = 585). The data are represented as frequency (N) and percentage (%). Percentages are calculated based on the total number of participants (N = 585). Data for the duration of eye symptoms before consulting a physician are presented as mean ± SD.

		Frequency (N)	Percentage (%)
The recommended time to start glaucoma screening is	After the age of 40 years	281	48.0%
	Before the age of 40 years	304	52.0%
Have you been screened for glaucoma before?	Yes	25	4.3%
	No	560	95.7%
Have you undergone an ocular examination/screening in the last year?	Yes	178	30.4%
	No	407	69.6%
		Mean	SD
Eye symptoms duration before consulting a physician (days)		4	2

Awareness level assessment

**Figure 1:**
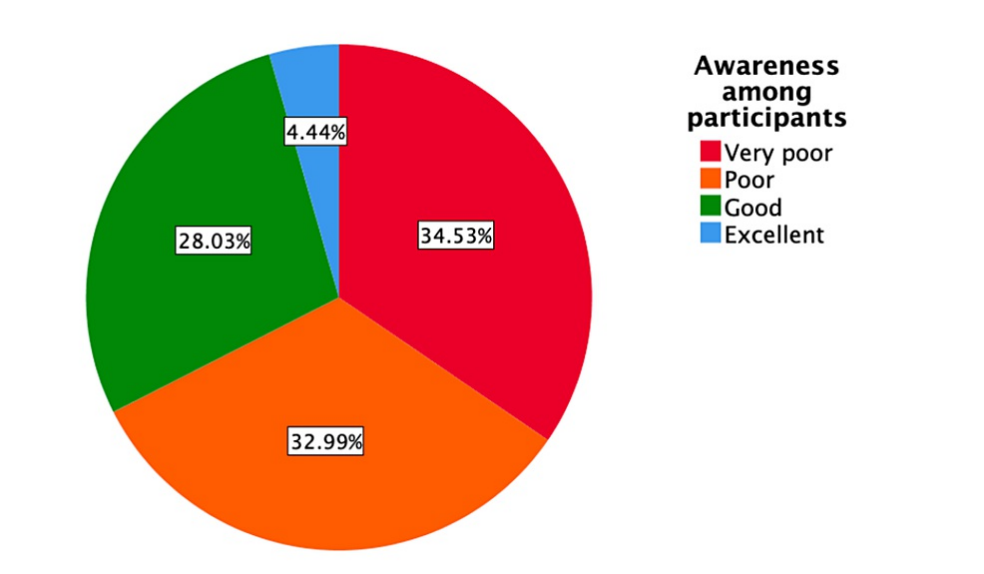
Glaucoma awareness among all participants. The data are represented as percentages (%). Percentages are calculated based on the total number of participants (N = 585).

**Figure 2:**
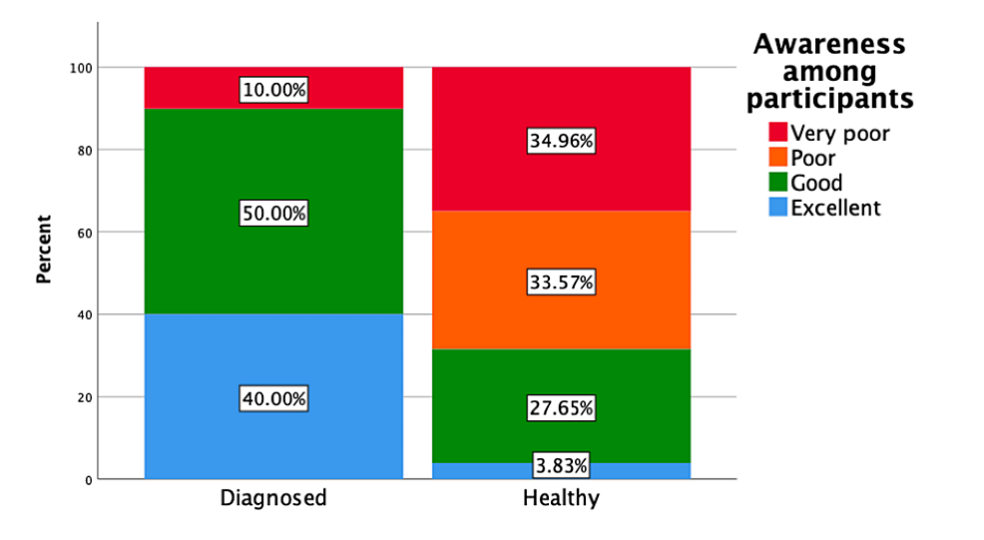
Glaucoma awareness in study participants who were (n = 10) and were not (n = 575) diagnosed with glaucoma. The data are represented as percentages (%). Percentages are calculated based on the total number of participants (N = 585).

Levels of awareness of glaucoma among all study participants are shown in Figure 1. Levels of awareness in study participants who had (n = 10) and had not (n = 575) been diagnosed with glaucoma are compared in Figure 2.

Distributions of awareness of glaucoma based on personal data and family histories of glaucoma are shown in Table 7. Awareness levels were significantly higher in women (p < 0.001) and in individuals diagnosed with glaucoma (p < 0.001) compared to men and those without a glaucoma diagnosis, respectively. Participants who reported family histories of glaucoma were more likely to exhibit good (n = 44, 46.8%) and excellent (n = 14, 14.9%) levels of glaucoma awareness than those without family histories of glaucoma (24.4% and 2.4%, respectively) (p < 0.001). Participants who reported that physicians were their main sources of information about glaucoma were more likely to exhibit good (n = 33, 42.3%) and excellent (n = 17, 21.8%) glaucoma awareness than those who reported that their main sources of information were social media (n = 60, 21.8% and n = 4, 1.5%, respectively) (p < 0.001). Awareness levels were significantly higher in individuals who had undergone glaucoma screening (p < 0.001) and those who had an ocular examination in the previous year (p = 0.001) compared to those who had not undergone these respective assessments.

**Table 7:** Glaucoma awareness levels in different subgroups of participants (N = 585). The data are represented as frequency (N) and percentage (%). Percentages are calculated based on the total number of participants (N = 585). The p-values were determined using Pearson’s chi-squared test, with statistical significance set at p < 0.05.

		Awareness level								
		Very poor		Poor		Good		Excellent		P-value
		Frequency (N)	Percentage (%)	Frequency (N)	Percentage (%)	Frequency (N)	Percentage (%)	Frequency (N)	Percentage (%)	
Age group (years)	18–25	154	36.2%	143	33.6%	111	26.1%	17	4.0%	0.587
	26–35	28	31.1%	27	30.0%	30	33.3%	5	5.6%	
	36–45	9	29.0%	11	35.5%	8	25.8%	3	9.7%	
	>45	11	28.2%	12	30.8%	15	38.5%	1	2.6%	
Gender	Male	129	41.7%	103	33.3%	67	21.7%	10	3.2%	<0.001
	Female	73	26.4%	90	32.6%	97	35.1%	16	5.8%	
Level of education	High school	49	44.1%	32	28.8%	27	24.3%	3	2.7%	0.136
	Bachelor’s degree	144	32.4%	154	34.7%	126	28.4%	20	4.5%	
	Masters and‎/or PhD	9	30.0%	7	23.3%	11	36.7%	3	10.0%	
Have you been diagnosed with glaucoma?	Yes	1	10.0%	0	0.0%	5	50.0%	4	40.0%	<0.001
	No	201	35.0%	193	33.6%	159	27.7%	22	3.8%	
Do you have a family history of glaucoma?	Yes	5	5.3%	31	33.0%	44	46.8%	14	14.9%	<0.001
	No	197	40.1%	162	33.0%	120	24.4%	12	2.4%	
What is your primary source of information on glaucoma?	Physician	15	19.2%	13	16.7%	33	42.3%	17	21.8%	<0.001
	Social media	115	41.8%	96	34.9%	60	21.8%	4	1.5%	
	Friends and‎/or relatives	56	30.4%	73	39.7%	52	28.3%	3	1.6%	
	Books and/or literature	16	33.3%	11	22.9%	19	39.6%	2	4.2%	
Have you been screened for glaucoma before?	Yes	2	8.0%	3	12.0%	12	48.0%	8	32.0%	<0.001
	No	200	35.7	190	33.9%	152	27.1	18	3.2%	
Have you undergone an ocular examination/screening in the last year?	Yes	47	26.4%	62	34.8%	53	29.8%	16	9.0%	0.001
	No	155	38.1%	131	32.2%	111	27.3%	10	2.5%	

## Discussion

This cross-sectional study investigated the awareness and knowledge of glaucoma in the adult population of Riyadh City, Saudi Arabia, as well as assessed associations between levels of glaucoma awareness and demographic factors such as age group and education level. To our knowledge, this is the first study to investigate awareness and knowledge of glaucoma in the general population of Riyadh City. These results may have significant public health consequences, as awareness of glaucoma in Riyadh City was found to be insufficient. Specifically, only 164 (28.0%) respondents had good awareness of glaucoma, with only 26 (4.4%) having excellent awareness. In contrast, 395 (67.5%) respondents had low levels of awareness, a percentage higher than the 52.4% of respondents in a cross-sectional study of visitors to ophthalmology clinics in Jordan [14], but lower than the 78.2% of respondents in Jeddah, Saudi Arabia [15]. The greater awareness of glaucoma in Jordan may be associated with the increased number of health education initiatives in that country. In contrast, the low levels of awareness of glaucoma in Jeddah and Riyadh populations may be due to their similar levels of general health education.

In this study, 221 (37.8%) participants had heard about glaucoma, compared with 73.9% in a survey in Mexico [16], 16.9% in a study at two family health centers in Istanbul [17], and 26.1% in a study in North India [18]. These differences may be attributed to regional differences in awareness campaigns, healthcare facilities, and public education programs, resulting in variations in glaucoma knowledge among study participants. Additionally, an extremely low percentage of participants in this study, 25 (4.3%), had previously undergone screening for glaucoma, similar to the percentages reported in a study of participants in Abha, Southern Saudi Arabia [12]. These low percentages may have been due to a lack of knowledge of the importance of glaucoma screening in both populations. Previous screening for glaucoma was also significantly associated with higher levels of glaucoma awareness in both Riyadh (p < 0.001) and Abha [12].

Of the 585 participants in this study, only 10 (1.7%) reported having been diagnosed with glaucoma. In contrast, 6.5% of the participants in a cross-sectional study conducted in Jazan, Saudi Arabia, had previously been diagnosed with glaucoma [19]. All participants in the latter study, however, were aged ≥40 years, whereas the majority of participants in the present study were aged 18-25 years. A diagnosis of glaucoma was significantly associated with the level of awareness of glaucoma, both in Riyadh and Jeddah [15].

A cross-sectional study of participants in Hail Province, Saudi Arabia reported that 14% of the participants had a family history of glaucoma, with family history being significantly associated with the level of glaucoma awareness [13]. These findings were consistent with the results of the present study, in which 16% of the participants reported having a family history of glaucoma, with family history being significantly associated with the level of glaucoma awareness (p < 0.001).

Consistent with previous findings [20,21], the present study found that participants who had undergone previous ocular examinations were more likely to exhibit better knowledge of glaucoma. Individuals who had undergone ocular examinations may have acquired information directly from ophthalmology specialists, may have been more proactive in acquiring information about eye health, and may have had access to instructional materials during those examinations.

The predominant source of information about glaucoma for most participants in this study was social media, aligning with the survey’s distribution platform. In contrast, most participants surveyed in Osun State, Southwest Nigeria, acquired information about glaucoma during visits to ophthalmology clinics [22]. A cross-sectional study in Germany found that friends and relatives were the main sources of glaucoma information [23], whereas a cross-sectional study in Damascus, Syria [24] found that 32.2% of the participants acquired glaucoma information from family, relatives, and friends. The present study found that the level of awareness of glaucoma information was significantly associated with the primary source of glaucoma information, consistent with the survey of participants in Abha, Saudi Arabia [12]. Of the participants in this study who received their information from physicians, 33 (42.3%) and 60 (21.8%) exhibited good and excellent levels of glaucoma awareness, respectively, indicating the benefits of deferring to an appropriately knowledgeable person for accurate information. Physicians, therefore, play an effective role in increasing the population’s awareness of glaucoma.

Several studies from around the world have reported links between higher education levels and better knowledge of glaucoma [25-27]. This was not evident in the present study, which found no significant association between education level and glaucoma knowledge, noting that the major contributors to this study were individuals between 18 and 25 years of age. In contrast to previous studies [28-30], the present study found that gender was significantly associated with glaucoma awareness (p = 0.001). These discrepancies may have been due to differences in study populations, methodologies, and/or cultural contexts. In addition, geographical differences, socioeconomic factors, and access to healthcare may have influenced glaucoma awareness.

This study had a few limitations. First, it did not include individuals who were illiterate or lacked Internet access. Only participants who could read, were accustomed to utilizing the internet, and had access to social media were able to complete the self-administered online questionnaire. Second, the use of a self-reported questionnaire may have introduced answer bias.

## Conclusions

The results of this study revealed a concerning lack of awareness regarding glaucoma among the adult population of Riyadh City, Saudi Arabia. The identified associations between awareness level and factors such as gender, family history of glaucoma, and primary source of information indicate the urgent need for comprehensive health education initiatives to enhance public awareness. These programs should be tailored to encompass the entire Saudi population, ensuring widespread dissemination of knowledge and understanding.
